# Comparison of efficacy of SHENQI compound and rosiglitazone in the treatment of diabetic vasculopathy analyzing multi-factor mediated disease-causing modules

**DOI:** 10.1371/journal.pone.0207683

**Published:** 2018-12-06

**Authors:** Hong Gao, Yuhong Duan, Xiaoxu Fu, Hongyan Xie, Ya Liu, Haipo Yuan, Mingyang Zhou, Chunguang Xie

**Affiliations:** 1 Teaching Hospital, School of Clinical Medicine, Chengdu University of Traditional Chinese Medicine, Chengdu, China; 2 Department Two of Endocrinology, Teaching Hospital, Shaanxi University of Traditional Chinese Medicine, Xianyang, China; Harbin Medical University, CHINA

## Abstract

Atherosclerosis-predominant vasculopathy is a common complication of diabetes with high morbidity and high mortality, which is ruining the patient's daily life. As is known to all, traditional Chinese medicine (TCM) SHENQI compound and western medicine rosiglitazone play an important role in the treatment of diabetes. In particular, SHENQI compound has a significant inhibitory effect on vascular lesions. Here, to explore and compare the therapeutic mechanism of SHENQI compound and rosiglitazone on diabetic vasculopathy, we first built 7 groups of mouse models. The behavioral, physiological and pathological morphological characteristics of these mice showed that SHENQI compound has a more comprehensive curative effect than rosiglitazone and has a stronger inhibitory effect on vascular lesions. While rosiglitazone has a more effective but no significant effect on hypoglycemic. Further, based on the gene expression of mice in each group, we performed differential expression analysis. The functional enrichment analysis of these differentially expressed genes (DEGs) revealed the potential pathogenesis and treatment mechanisms of diabetic angiopathy. In addition, we found that SHENQI compound mainly exerts comprehensive effects by regulating MCM8, IRF7, CDK7, NEDD4L by pivot regulator analysis, while rosiglitazone can rapidly lower blood glucose levels by targeting PSMD3, UBA52. Except that, we also identified some pivot TFs and ncRNAs for these potential disease-causing DEG modules, which may the mediators bridging drugs and modules. Finally, similar to pivot regulator analysis, we also identified the regulation of some drugs (e.g. bumetanide, disopyramide and glyburide etc.) which have been shown to have a certain effect on diabetes or diabetic angiopathy, proofing the scientific and objectivity of this study. Overall, this study not only provides an in-depth comparison of the efficacy of SHENQI compound and rosiglitazone in the treatment of diabetic vasculopathy, but also provides clinicians and drug designers with valuable theoretical guidance.

## Introduction

Diabetes is a metabolic disease characterized by hyperglycemia caused by impaired insulin secretion or impaired function. When patients with hyperglycemia for a long time, their various tissues, especially the eyes, kidneys, heart, blood vessels and nerves, are chronically damaged, which resulting in dysfunction. Diabetic macroangiopathy is typically atherosclerosis with higher morbidity and mortality caused by hyperglycemia, and the progression is deteriorating quickly and seriously. About 70% to 80% of diabetic patients die of diabetic vasculopathy, which arouses the attention of urologist clinicians and drug developers. Currently, it is considered that advanced glycation end products (AGEs), the defect of signal transduction and the imbalance of matrix metalloproteinase are important inducement of coronary artery sclerosis and cerebral atherosclerosis[1]. AGEs play an important role in diabetic vascular complications. Oxidative stress reaction will induce AGEs interact with their receptors (RAGE), triggering inflammation and thrombosis[2]. In addition, Migdalis IN *et al*. showed that there is a positive correlation between fasting insulin levels and endothelin that as a marker of atherosclerosis[3]. *Lu JP et al*. showed that in hyperglycosemia environment, calcium-sensitive receptors (CaSRs), typical G protein-coupled receptors, can active caspase-3 and -9, Bax, phospho-p38 and phosphor-JNK to apoptosis, which causes macrovascular disease [4]. Nelaeva AA *et al*. found that the sustained decrease of interleukin-8 (IL-8) resulting from angiogenesis is one of the factors of vascular lesions in diabetic patients [5]. These studies showed that hyperglycosemia has different degrees of influence on various pathways and physiological processes in the blood vessels, which eventually inducing atherosclerosis and severely damaging blood vessels, predicating the control of blood glucose and the improvement of insulin resistance are of great significance in prevention and treatment of diabetic macrovascular disease [6].

In medical treatment, western medicine such as pioglitazone and metformin are used to exquisitely regulate glucose and lipid metabolism by increasing the sensitivity of insulin, which precisely control blood glucose concentration and play an anti-atherosclerosis characteristics [7]. While Chinese medicine compound, as the essence of traditional Chinese medicine (TCM), has good therapeutic efficacy for diabetes and the macroangiopathy complications. For example, MaiTong compound (MTF) effectively treats spontaneous diabetic macroangiopathy by modulating the key proteins involved in the AMPK signaling pathway and vascular smooth muscle contraction [8]. Liu Y *et al*. showed that SHENQI compound can inhibit angiogenesis and prevent diabetic macroangiopathy by promoting the expression of PTEN in the aortic wall and inhibiting the expression of PI3Kp85 in the aorta [9]. Our previous study also found that SHENQI compound, with the function of yangyinyiqi and huoxue, can activate insulin target organs and inhibit PI3-K signaling pathways in non-target organs in the process of preventing and treating diabetic macroangiopathy, indicating the significant tissue specificity and the integrity of SHENQI compound.

Therefore, to compare the therapeutic efficacy of TCM and western medicine on diabetic angiopathy, we selected two typical drugs for the treatment of diabetic vasculopathy, Chinese medicine SHENQI compound and western medicine rosiglitazone to conduct systematic research. It aimed to analyze and compare the preventive and therapeutic effects of SHENQI compound and western medicine rosiglitazone on diabetic angiopathy, and to reveal the pathogenesis of diabetes and its vascular complications. The scientific and systematic analysis not only has important reference and corroboration effect on the comparison of two drug treatment mechanisms, but also has valuable guiding significance for clinicians to personalize according to different conditions.

## Results

### Behavior, physiological and pathological changes in mice after drug treatment

To compare and analyze the effect and mechanism of TCM SHENQI compound and western medicine rosiglitazone on diabetic angiopathy, we built seven groups of model mice, including spontaneous diabetic group (group A), SHENQI compound group (group B), diabetic macroangiopathy group (group C), yangyinyiqi group (group D), huoxue group (group E), rosiglitazone group (group F) and healthy C57 mouse control group (group G). In behavioral performance, mice with diabetes mellitus (A) and diabetic macroangiopathy (C) were irritated, bit each other and gradually became apathetic, unresponsive and slow-acting with the age increasing. The irritable mood and the bit were the most serious in the rosiglitazone group (F) and the diabetic macroangiopathy group (C). The SHENQI compound group (B) was shiny, no scratches, relatively gentle temperament and better mental state. However, the scratching marks and wounds on the body surface were observed both in the yangyinyiqi group (D) and the huoxue group (E), and the conditions in the group D were more serious.

In physiological indicators (S1 Table), with the age increasing, blood glucose gradually increased in diabetic group (A). In the rosiglitazone group (F), the hypoglycemic was observed in 3th week and significantly different from group A. Although the hypoglycemic curve showed a downward trend, the fluctuation of blood glucose was larger. In the SHENQI compound group (B), the hypoglycemic was observed in 5th week and significantly different from group A. And the hypoglycemic effect was weaker than that of group F. The hypoglycemic effect of yangyinyiqi group (D) and huoxue group (E) were weaker than that of SHENQI compound group (B), but there is no significant difference among the three.

**Fig 1:**
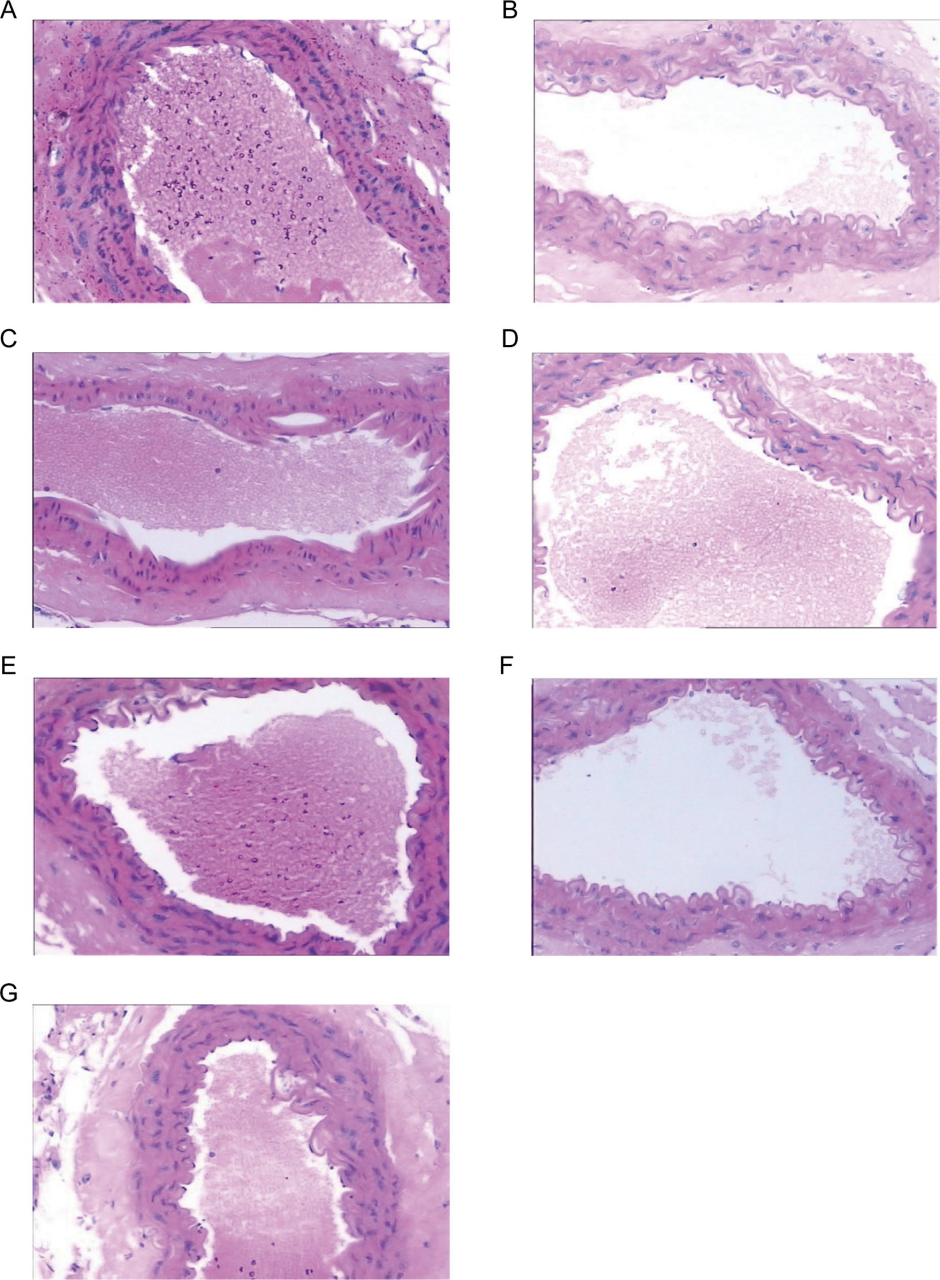
Pathological morphology comparison. (A) Images of abdominal aortic intima pathology in healthy C57BL/6J mice. The vascular endothelial cells are flat and closely attached to the flat inner elastic plate. The medium elastic plate and the vascular smooth muscle cells are arranged in parallel and the inner membrane is smooth and tidy. (B) KKAy Pathological section imaging of vascular endothelial cells in spontaneous diabetic mice. The vascular endothelial cells showed swelling and no obvious endothelial cell shedding. (C) Pathological section imaging of abdominal aortic endothelial cells in diabetic macroangiopathy group. The abdominal aortic endothelial cells showed high edema, large area and continuous flaking, and the intima was not smooth. (D) Pathological section imaging of abdominal aortic endothelial cells in mice in the rosiglitazone group. It appears to be scattered in focal endothelial cells with mild edema. (E) Pathological section imaging of abdominal aortic endothelial cells in the SHENQI compound group. Occasionally, it is spotted and accompanied by mild edema. (F) Images of pathological sections of abdominal aortic endothelial cells in mice of yangyinyiqi group. (G) Pathological section imaging of abdominal aortic endothelial cells in mice of huoxue group. The vascular endothelial cells of the yangyinyiqi group and the huoxue group showed cell shedding and edema, which was lighter than that of the diabetic group and more severe than the SHENQI compound group.

In terms of pathological sections, the abdominal aorta intima was flat in the healthy mice (G), the vascular endothelial cells were flattened and adhered to a flat inner elastic plate, the bullet plate and vascular smooth muscle cells in parallel arrangement and the inner membrane was smooth and tidy (Fig 1A). In spontaneous diabetic group (A), vascular endothelial cells were swollen, and endothelial shedding was not obvious (Fig 1B). In diabetic macroangiopathy group (C), the abdominal aortic endothelial cells were highly edematous, large areas and sustained flake, and the intima was not smooth (Fig 1C). The rosiglitazone group (F) showed scattered shedding of focal endothelial cells with mild edema (Fig 1D). The endothelial cells of SHENQI compound group (B) were slightly deciduous and mild edema (Fig 1E). And yangyinyiqi group (D) and huoxue group (E) showed cell shedding and edema, which was lighter than that of diabetic group (A) and more severe than SHENQI compound group (B) (Fig 1F and 1G).

### Differential expression analysis

**Fig 2:**
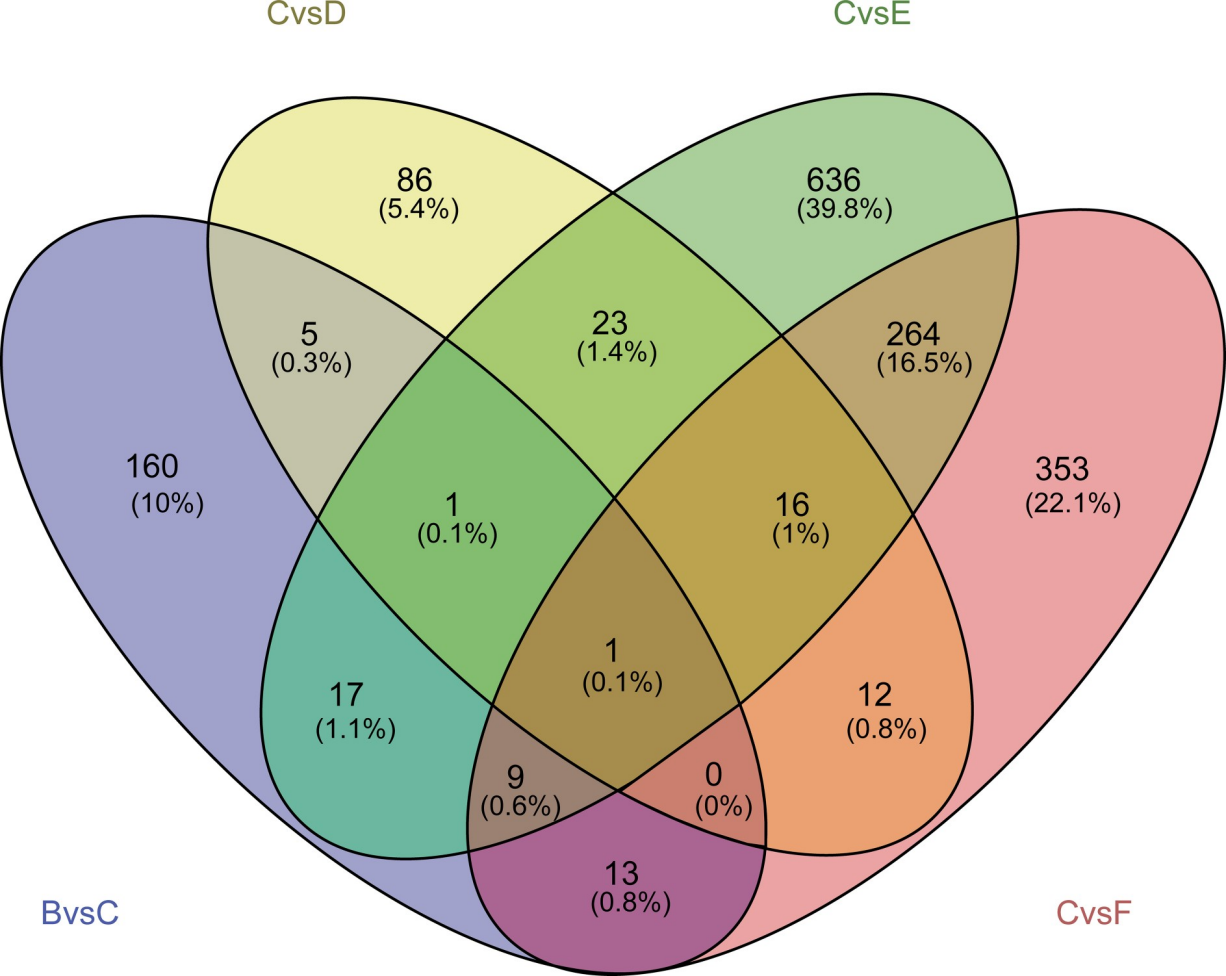
Differentially expressed genes in SHENQI compound group (blue), yangyinyiqi group (yellow), huoxue group (green) and rosiglitazone group (red).

**Fig 3:**
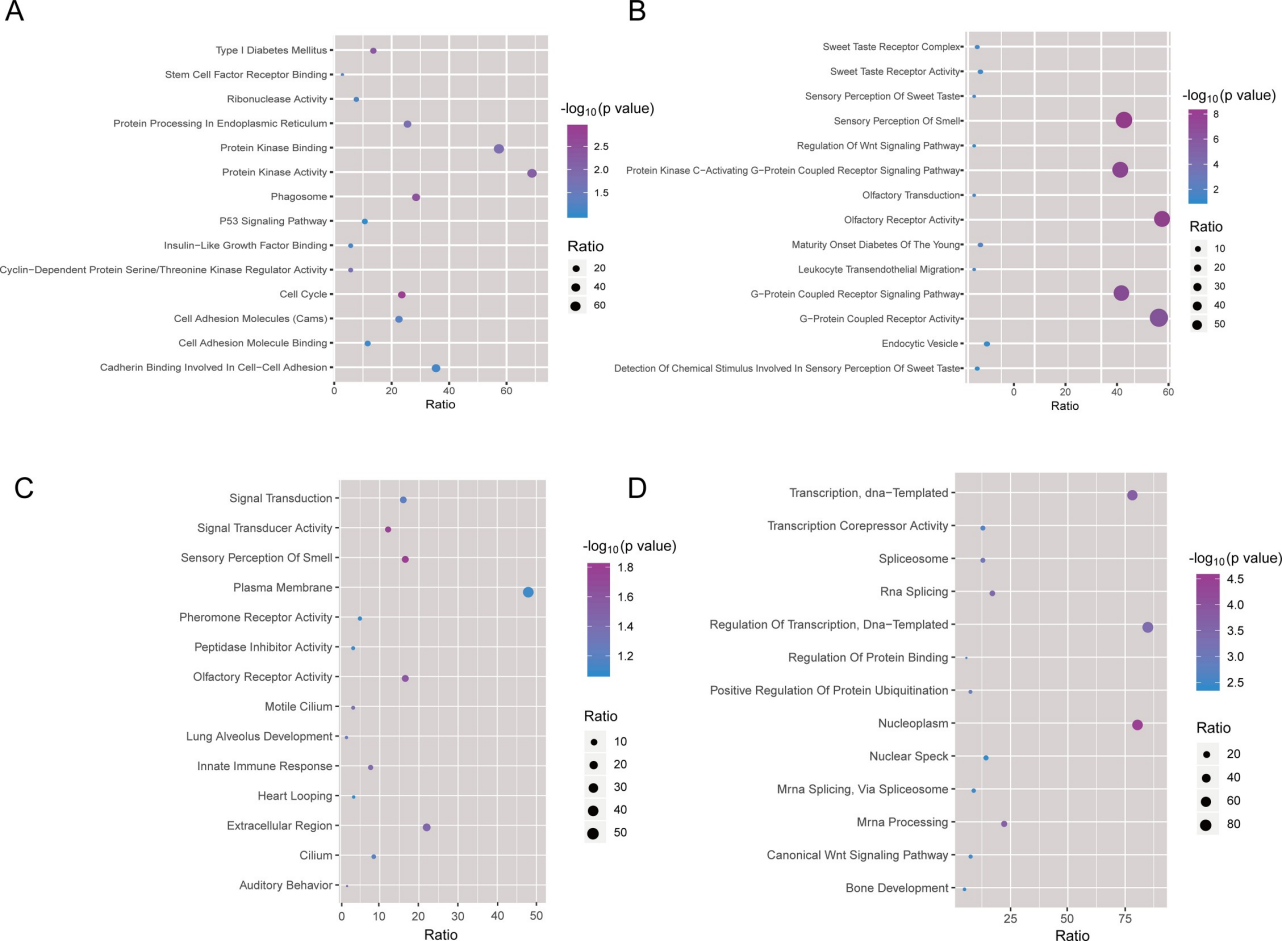
Functional and pathway enrichment analysis of differentially expressed genes. (A) The functions and pathways differentially expressed genes (diabetic mice VS normal mice) involved in. (B) The functions and pathways differentially expressed genes (diabetic vascular lesions VS diabetic mice) involved in. (C) The functions and pathways differentially expressed genes in SHENQI compound group involved in. (D) The functions and pathways differentially expressed genes in rosiglitazone group involved in.

Behavior, physiology and pathological changes are important manifestations of gene expression changes. Exploring differentially expressed genes in different condition and the functions and pathways they involved in are important for revealing the pathogenic mechanisms and therapeutic mechanisms. Here, we identified differentially expressed (DE) genes for these group, respectively (Fig 2, S2 Table). Functional enrichment showed that DE genes in diabetes were mainly involved in the imbalance of sweetness and olfaction, protein kinase C-activated G protein-coupled receptor signaling pathways, young adult-onset diabetes mellitus and regulation of Wnt signaling pathways (Fig 3, S3 Table). DE genes in diabetic macroangiopathy group were mainly enriched in cadherin binding in the intercellular adhesion, and positive regulation of cell adhesion. The dysfunction of these genes may eventually induce vascular lesions, for example atherosclerosis.

In addition, functional enrichment analysis of DE genes in SHENQI compound and rosiglitazone group suggested that SHENQI compound mainly stabilizes the patient's emotions by regulating sensory perception and the central nervous system, lowers blood glucose by modulating immune responses and polysaccharide synthesis, and inhibits the progression of vascular lesions by modulating G protein-coupled receptor signaling pathways. While rosiglitazone lowers blood glucose rapidly by impacting the Wnt signaling pathway, reverse regulation of the JAK-STAT cascade and heparin sulfate proteoglycan biosynthetic methods. Moreover, it modulates the response to interleukin-18, cytokine-mediated signal transduction pathways and immune responses such as T cell-mediated positive regulation of cytotoxicity, which has a significant effect on type I diabetes. Rosiglitazone also inhibits vascular lesions through regulating cell adhesion. However, compared to diabetes, the effect of rosiglitazone on the treatment of vascular lesions is not significant.

### Identifying disease-causing modules and pivot regulators

**Fig 4:**
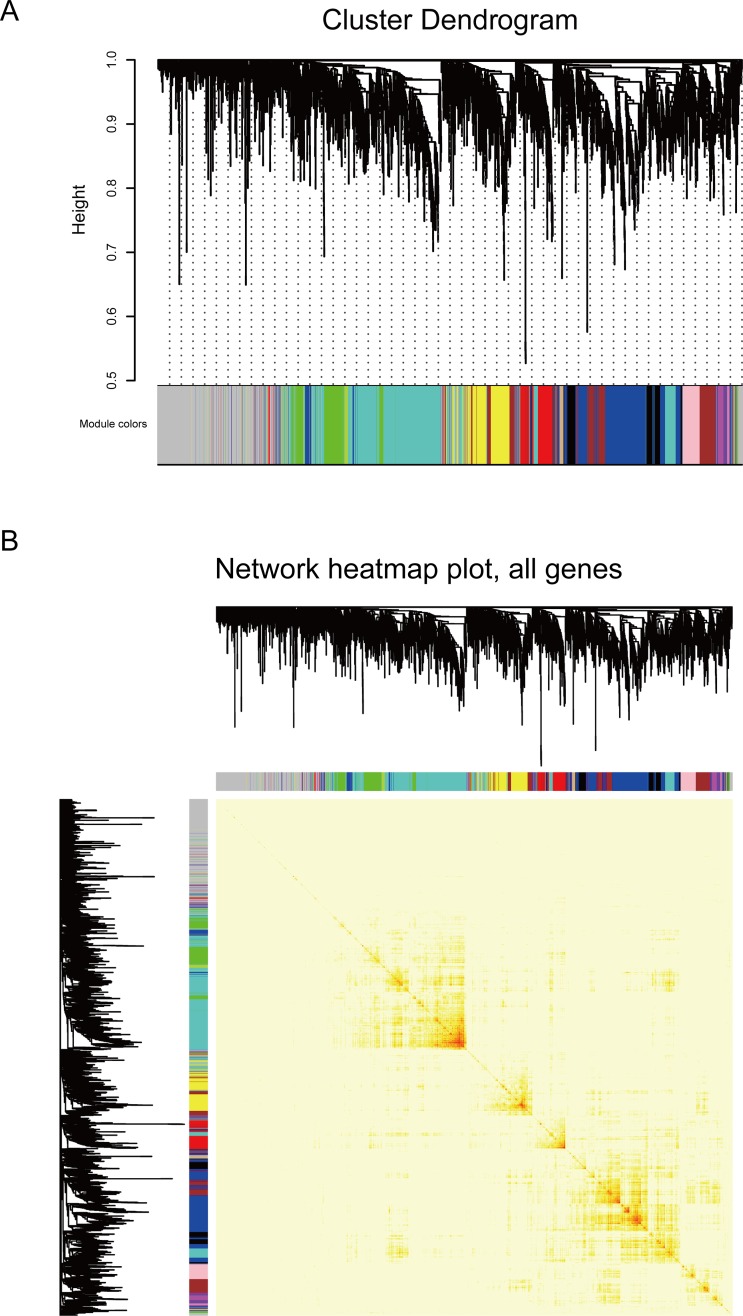
Co-expression analysis for mining function module. (A) Clustering dendrogram of genes. A total of 13 colors corresponding to 13 modules. X-axis represents gene and y-axis represents the height of the gene tree. (B) Heat map of the expression of the module gene. Light color represents low overlap and progressively darker red color represents higher overlap. Blocks of darker colors along the diagonal are the modules.

**Fig 5:**
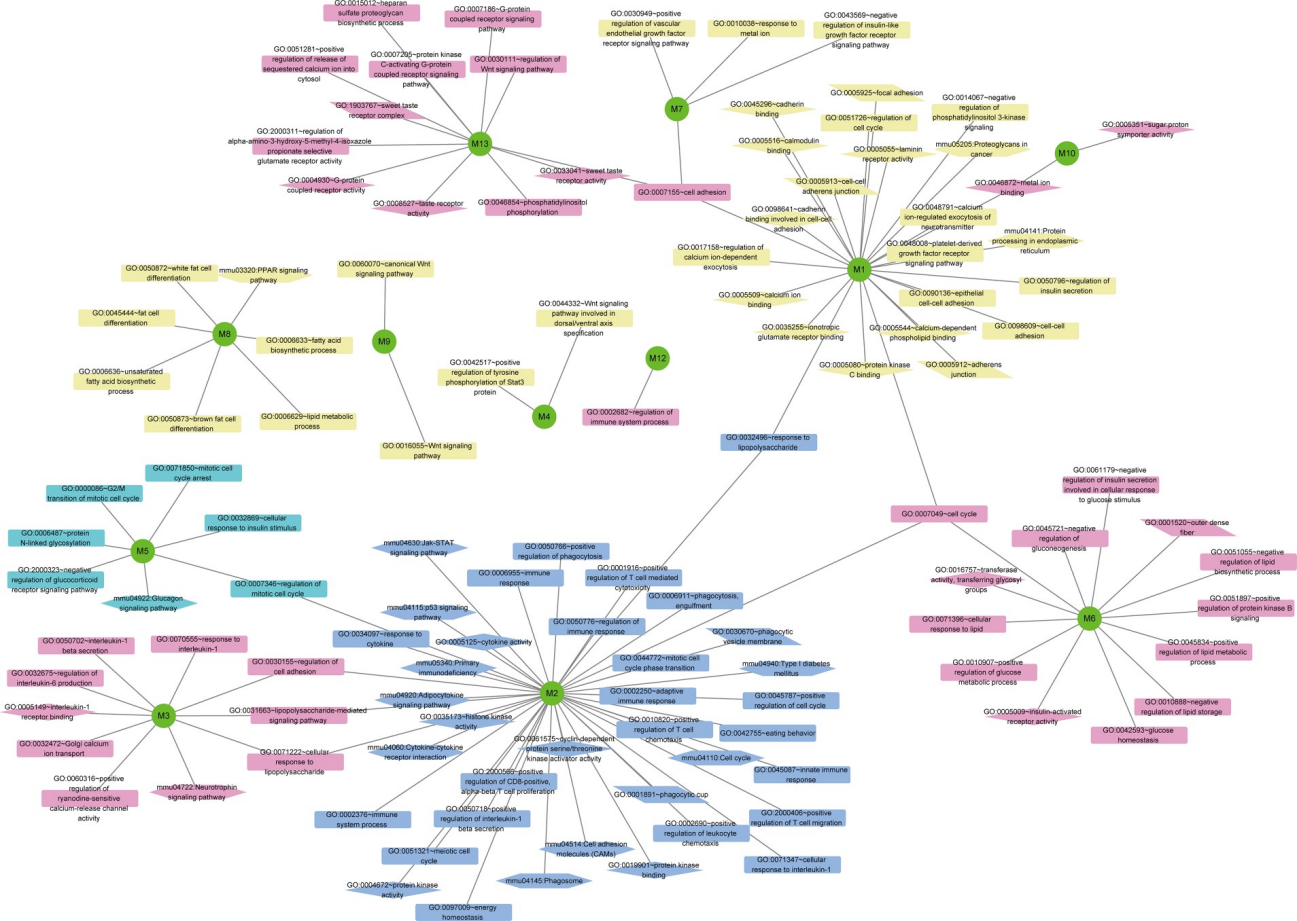
Functional and pathway enrichment of potential disease-causing modules. The green circle represents module, the rectangle represents the biological process of the GO function, the diamond represents the molecular function of the GO function, the parallelogram represents the cellular composition of the GO function, and the hexagon represents the KEGG pathway. Blue represents a type I diabetes-related functional pathway, cyan represents a type II diabetes-related functional pathway, yellow represents a major vascular disease-related functional pathway, and red represents a diabetic vascular lesion complex pathway.

Understand the pathogenesis of disease is essential for revealing the mechanism of drug treatment. Here, we explored co-expression modules based on the DE genes identified from spontaneous diabetic group and diabetic macroangiopathy group and 13 modules were exacted (Fig 4). And according to functions and pathways module genes involved in, modules were defined as potential disease-causing modules (Fig 5, S4 Table). Functional enrichment analysis showed that module 2 was mainly involved in positive regulation of T cell-mediated cytotoxicity, innate immune response a type I diabetes, which was identified as a potential type I diabetes-causing module. Module 5 was mainly involved in regulating insulin-stimulated cellular responses, negative regulation of the glucocorticoid receptor signaling pathway and insulin resistance-related functions and pathways, which was identified as a potential type 2 diabetes-causing module. Module 1, 4, 7, 8 and 9 were involved in intercellular adhesion due to cadherin binding, positive regulation of tyrosine phosphorylation of Stat3 protein, negative regulation of insulin-like growth factor receptor signaling pathway, PPAR signaling pathway and Wnt signaling pathways, which were identified as potential vascular disease-causing modules. Module 3, 6, 10 and 13 were identified as complex disease-causing modules, which were involved in both diabetes-related functions and pathways and the physiological processes of vascular lesions.

**Fig 6:**
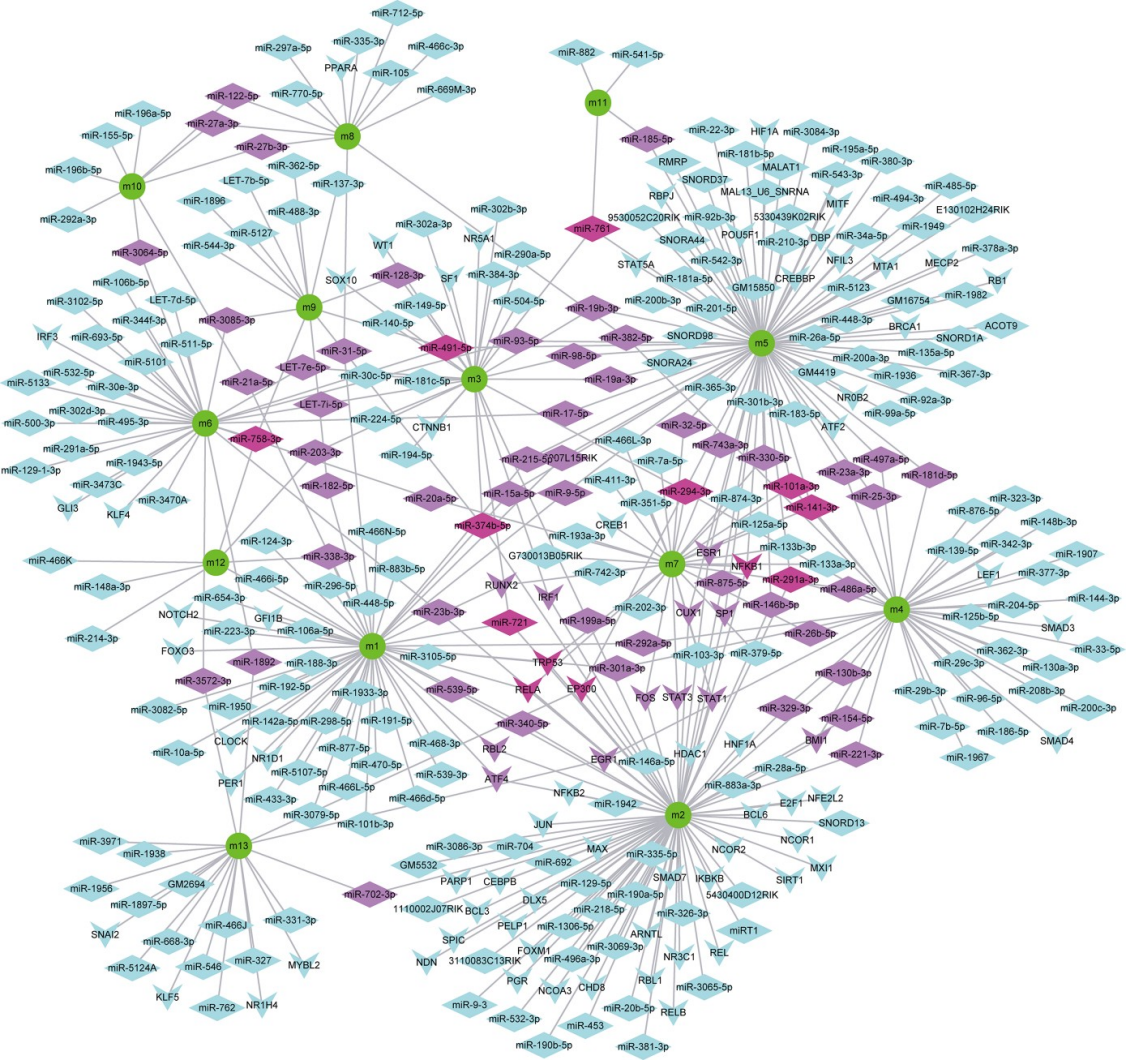
Regulation of ncRNAs and TFs on potential disease-causing modules. The green circle represents the potential disease-causing module, the diamond represents ncRNA, and the arrow shape represents the transcription factor (TF). The color from red to cyan represents the number of modules regulated by regulators (ncRNAs and TFs).

In addition, we identified pivot regulators for these potential disease-causing modules (Fig 6) based on TF-target and ncRNA-associated interactions, including 83 TFs (S5 Table) and 256 ncRNAs (S6 Table). We found that some of these have been validated associated with diabetes. For example, EP300 can inhibit histone deacetylase (HDAC) to promote the development of diabetes [10]. NFKB1 can increase the susceptibility of type 2 diabetes and kidney disease [11,12]. RELA can suppress inflammation and improve insulin sensitivity, which significantly regulate type I and type II diabetes [13,14].MiR-101a can promote inflammatory cytokine-mediated beta-cell dysfunction to regulate the development and progression of type I diabetes [15]. MiR-141 can regulate the expression of islet transcription factors to control the differentiation of insulin-producing cells (IPCs) [16]. These pivot regulators play an important role in manipulating these potential disease-causing modules. Since regulatory factors can act to mediate disease progression by activating multiple potential pathogenic modules, the therapeutic strategies based on regulatory factors of drug targets can achieve more comprehensive and effective therapeutic effects.

### Mechanism of SHENQI compound and rosiglitazone

**Fig 7:**
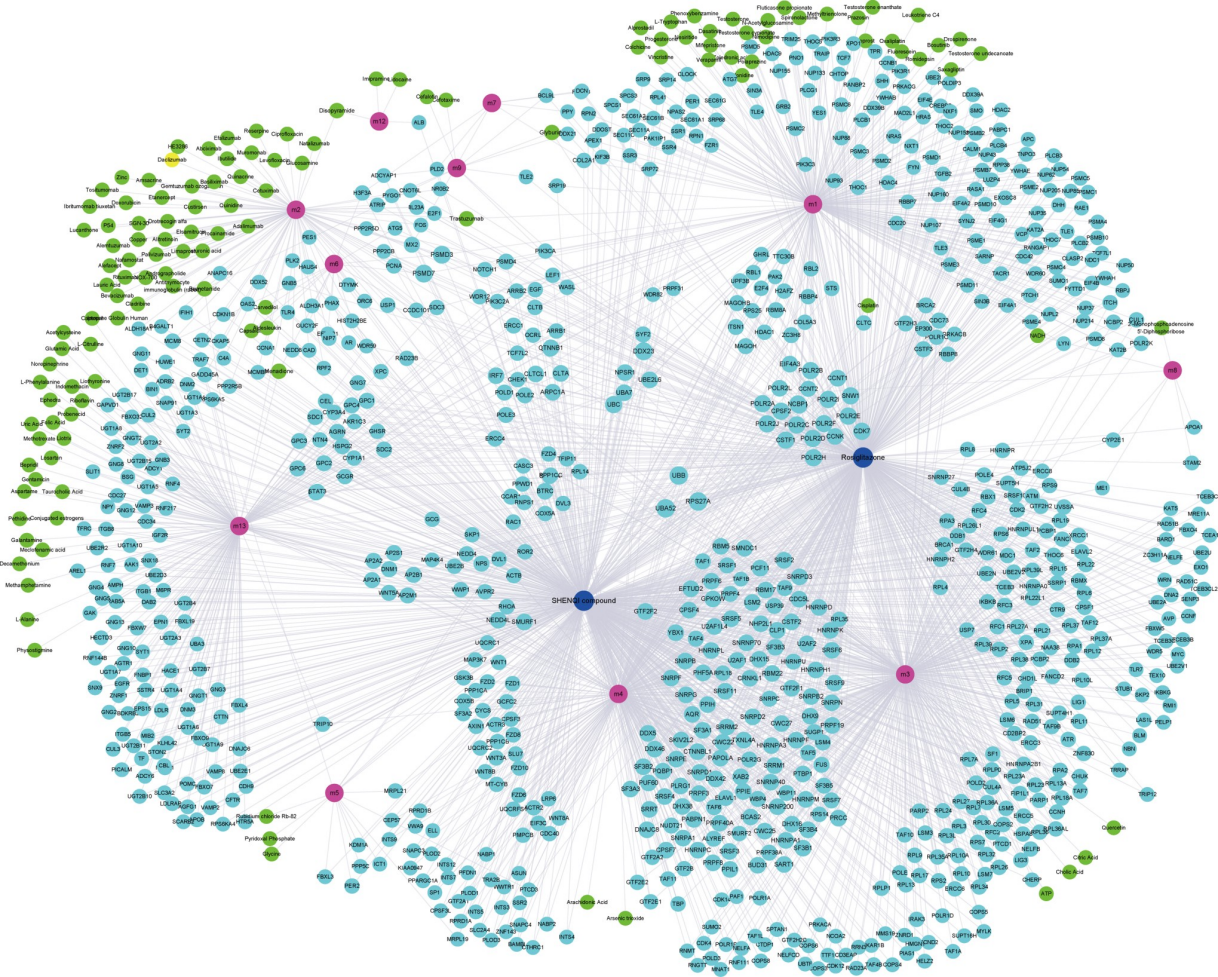
The significant relationships between drugs and modules. The red circle represents the module. The blue nodes represent the SHENQI compound and rosiglitazone and the green circle represent other potential drugs. The cyan node represents the potential targets of SHENQI compound and rosiglitazone.

Based on multi-factor mediated potential disease-causing modules and drug-target information, we identified potential drug for diabetes, including SHENQI compound and rosiglitazone (Fig 7, S7 Table). SHENQI compound targeted 688 genes. Among them, interferon regulatory factor 7 (IRF7) is a new strategy for the treatment of obesity and type 2 diabetes [17]. NEDD4Lis a key gene that associated with type 2 diabetes and cardiovascular continuous complications represented by ischemic heart disease (IHD) [18]. In addition, CDK7 is regulated by glucose in renal cells, which may become a potential target for the treatment of diabetic [19]. MCM8 has been shown to have a significant regulatory relationship with the prognosis and survival of pancreatic cancer [20]. Rosiglitazone targeted 613 genes, among them, PSMD3 is involved in regulating insulin signaling to mediate insulin resistance [21]. UBA52 is a diagnostic marker for diabetes [22], which may be a potential therapeutic target. PSMD3, a subunit of RNA polymerase, can induce the expression of Cox2, S100A4/FSP-1 and vimentin genes in renal, which contributes to diabetes-mediated chromatin turnover and promotes transcriptional changes in diabetic kidneys [23].

In addition to SHENQI compound and rosiglitazone, bumetanide, disopyramide, glyburide, menadione, trastuzumab and other drugs also were identified (Fig 7, S8 Table). Bumetanide is a commonly used drug for the treatment of diabetes and plays an important role in vascular homeostasis and inflammation [24], suggesting it may be an effective treatment for diabetic vasculopathy. While disopyramide, glyburide, menadione and trastuzumab have been used in clinical diabetes treatment. These strongly support the scientific and objectivity of the potential disease-causing module theory in the study used to explore SHENQI compound and rosiglitazone drug therapy mechanism.

In addition, we also observed the possibility of pivot TFs as drug targets. By observing the differential expression of pivot TFs in the diabetic macroangiopathy model group and the corresponding treatment group, we identified the yangyinyiqi component of SHENQI compound to target PER1, while the huoxue component can target SMAD7 and DBP to reconcile potential pathogenic modules to inhibit disease progression. On the other hand, NFE2L2, MTA1, NR0B2, RB1 and MYBL2, which were potential targets of rosiglitazone, resulting in significant therapeutic efficacy.

## Discussion

At present, the treatment and research of diabetes and its complication are mainly based on blood sugar, but evidence indicates that the enhancement of hypoglycemic is not effective in reducing the incidence of the terminal event of large vessels. The clinical application of TCM SHENQI compound for 15 years has definite curative effect on preventing and treating diabetic macroangiopathy, and it also shows the function that cannot be achieved during the treatment of western medicine [9, 25, 26]. To systematically compare the treatment mechanisms of Chinese and Western medicines and emphasize the more significant and more comprehensive therapeutic effects of SHENQI compound, we performed a series of experiments and analysis. Results showed that mice treated with different drugs showed significant differences in behavioral performance, while similar in the physiological indicators and pathological morphology.

We then performed differential expression analysis and functional enrichment analysis. Related genes were mainly involved in immune system processes and inflammatory responses, which may be a potential mechanism of vascular endothelial cell swelling. In addition, they also regulated lipopolysaccharide-mediated signaling pathways, fatty acid catabolism and other functional pathways, causing abnormalities in physiological indicators. Autophagic cell death, negative regulation of G1/S transition of mitotic cell cycle and other autophagy and cell cycle-related signaling pathways are abnormal when diabetic mice develop macrovascular disease. As a result, the endothelial cells are severely edematous, and large-scale and continuous flaking occurs, causing the endometrium to exhibit a smooth condition. At the same time, the dysregulation of glycolipid metabolic signaling pathways such as gluconeogenesis, acetylglucosaminyl transferase activity, lipopolysaccharide-mediated signaling pathway, etc., increased the levels of carbohydrates and lipids in plasma. Moreover, SHENQI Compound and rosiglitazone can target and regulate related genes, thereby balancing the functions and pathways of these disorders to suppress and alleviate the corresponding symptoms. In addition, SHENQI compound has a significant regulatory effect on sensory perception such as olfaction and central nervous system, which helps to stabilize the patient’s mood, it also inhibits the development of vascular lesions by regulating the G protein-coupled receptor signaling pathway [4] and the positive regulation of phosphatidylinositol 3-kinase activity [9]. Rosiglitazone is involved in the Wnt signaling pathway, reverse regulation of the JAK-STAT cascade and heparin sulfate proteoglycan biosynthetic methods, which associated with the regulation of blood glucose 9[27–29]. In a word, SHENQI compound has a more comprehensive therapeutic effect, which not only effectively reduces the patient's blood glucose, but also has a significant inhibitory effect on vascular lesions. Moreover, the regulation of sensory and central system makes it effective in the clinical treatment of excitable patients. While the main role of rosiglitazone is to rapidly lower blood glucose and to reduce the incidence of vascular lesions.

To investigate the therapeutic mechanisms of SHENQI compound and rosiglitazone in depth, we identified 13 potential disease-causing modules and pivot regulators for these modules. The relationships between modules and pivot regulators may play an essential role in the occurrence and development of diabetic vasculopathy. For example, miR-101a can promote inflammatory cytokine-mediated beta-cell dysfunction, which influences the development and progression of type I diabetes [15]. MiR-141 has been reported is an important regulator of angiopathy and progressive renal disease [30,31]. Ke B *et al*. showed that APS-regulated miR-721-PPAR-γ-PI3K/AKT-GLUT4 signaling pathway attenuates TNF-α-induced insulin resistance [32], emphasizing the role of miR-721 in the regulation of diabetes and suggesting miR-721 may be potential drug target for SHENQI compound. Transcription factor EP300 can inhibit histone deacetylase (HDAC) to limit the development of pancreatic β-cells and α-cells, which influencing the development of diabetes [10,33]. NFKB1 can increases the susceptibility of type 2 diabetes and kidney disease [11,12]. Klotho-regulated serine (RELA) phosphorylation can negatively regulate the production of NF-κB-linked inflammatory proteins and upregulate the cAMP/PKA pathway, which suppressing inflammation and improving insulin sensitivity, significantly regulating type I and type II diabetes [13,14]. In conclusion, these pivot regulators mediating these potential disease-causing modules have important regulatory effects on diabetes and its complications of vascular disease, which may be potential drug target.

Further, we explored the therapeutic mechanism of SHENQI compound and rosiglitazone based on these potential disease-causing modules and drug-target information. The results showed that SHENQI compound can regulate the disease-causing module by targeting genes such as MCM8, IRF7, CDK7 and NEDD4L to exert comprehensive efficacy. MCM8 is involved in the regulation of DNA synthesis, regulating cell division in S phase, and has been shown to have a significant regulatory relationship with the prognosis and survival of pancreatic cancer [20]. Interferon regulatory factor 7 (IRF7) is identified as a novel cardiovascular stress inducer in pathologically stressed hearts and involved in the etiology of metabolic disorders, which becoming a new strategy for the treatment of obesity and type 2 diabetes [17,34]. CDK7, a putative thiol-related gene, is regulated by glucose in renal cells and exerts oxidative stress, which may become a potential target for the treatment of diabetic nephropathy [19]. NEDD4L is associated with type 2 diabetes and cardiovascular continuous complications represented by ischemic heart disease (IHD) [18,35]. While rosiglitazone mainly acts to rapidly lower blood sugar by targeting genes in modules, incluingPSMD3, UBA52 and POLR2A, etc. PSMD3 is involved in regulating insulin signaling to mediate insulin resistance and regulated by diet [21]. Ubiquitin ribosomal fusion protein (UBA52) is detected in urine of patients with diabetes, which is identified as a diagnostic marker for diabetes [22] and may be a potential therapeutic target. POLR2A, a subunit of RNA polymerase II (Pol II) can induce the expression of Cox2, S100A4/FSP-1 and vimentin genes in renal, which contributes to diabetes-mediated chromatin turnover and promotes transcriptional changes in diabetic kidneys [23].

The comparative analysis gives us an in-depth understanding of the therapeutic mechanisms of SHENQI compound and rosiglitazone. However, whether the disease-causing module theory is scientific? In addition to SHENQI compound and rosiglitazone, we also identified other drugs significantly associated with these modules. For example, bumetanide is a commonly used drug for the treatment of diabetes. It plays an important role in vascular homeostasis and inflammation [24]. Bumetanide is also an effective treatment for diabetes and vascular lesions. It has shown that drugs such as disopyramide, glyburide, menadione, etc. also have the function of regulating blood glucose and have been used for clinical treatment [36–38]. Trastuzumab has also been used to treat diabetes, but it has potential cardiotoxicity that is not conducive to reducing cardiovascular risk [39]. These suggest the investigation of the treatment mechanism based on the potential disease-causing modules is scientific and objective.

In addition, we have focused on the regulation of drugs on pivot TF to achieve a more comprehensive therapeutic effect. PER1, which has been verified to participate in the regulation of circadian rhythm, target by the yangyinyiqi component of SHENQI compound to mediates diabetes [40]. The huoxue component can inhibit TGF-β1 signaling by targeting SMAD7 to regulate the establishment and maintenance of mature pancreatic β cells, which is closely related to reversible diabetes [41]. On the other hand, rosiglitazone can not only activate NFE2L2 to protect cells from oxidative stress, but also play an important role in the prevention and treatment of diabetes and its complications [42]. And rosiglitazone can regulate the phosphatidylinositol 3-kinase/Akt signaling pathway and inhibit NPY gene expression in the hypothalamus though promoting the activation of RB1, which significantly reducing fasting blood glucose and improving glucose tolerance, have positive significance for the clinical treatment of diabetes [43].

In summary, this study developed a multi-factor mediated disease-causing modules map to deeply analyze the therapeutic mechanism of SHENQI compound and rosiglitazone. The two drugs showed significant differences in the treatment mechanisms and achieved different therapeutic effects. SHENQI compound has obvious advantages in stabilizing patient's mood and comprehensively treating complications of vascular lesions, while rosiglitazone has a great advantage in lowering blood sugar, but this advantage is relatively insignificant when compared with SHENQI compound. In addition, the relationships between drugs and pivot regulators still need more in-depth experiments to explore [44–47]. This will serve as our future research direction, aiming to comprehensively and in-depth explore and compare the therapeutic mechanisms of TCM SHENQI compound and Western medicine, and lays a theoretical foundation for the design and development of new drugs.

## Materials and methods

### Mice model and medicines

In this study, 90 male spontaneous type 2 diabetic KK/Ay mice with two random blood glucose levels ≥13.9 mmol/L and 15 male healthy mice aged C57BL/6 were tested, which aging 18-20-week-old. All mice were provided by the Experimental Animal Center of the Chinese Academy of Medical Sciences and Beijing Huayekang Biotechnology Co., Ltd. Seventy-five KK/Ay mice were randomly selected and daily drinking water and high-fat diet containing the nitric oxide synthase (NOS) inhibitor L-NAME (0.2 mg/ml) were given for 8 weeks to establish a model of diabetic macroangiopathy. The seventy-five mice were ranked according to their blood glucose levels from high to low, and they were randomly divided into diabetic macroangiopathy model group, SHENQI compound group, yangyinyiqi group, huoxue group and rosiglitazone group. The remaining 15 KK/Ay mice and 15 C57BL/6 healthy mice were classified as spontaneous diabetes group and C57 healthy group, respectively. Each group is processed according to the corresponding conditions (Table 1) for 8 weeks and observing the behavioral and histopathological features of each group of mice were observed, recording blood glucose, lipids and other physiological indicators. In addition, the skeletal muscle tissues of 3 mice were randomly collected in each group about 0.2–0.5g, and the aorta was about 0.1–0.3G. Subsequently, the sample RNA was extracted and the RNA level of all the samples was detected by Agilent 4*44k full expression microarray. In this experiment, all Chinese herbs were produced by the Department of Pharmacy, Affiliated Hospital of Chengdu University of Traditional Chinese Medicine, and rosiglitazone was produced by GlaxoSmithKline. All experimental procedures were approved by the Institutional Animal Care and Use Committee of Chengdu University of Traditional Chinese Medicine.

**Table 1:** Processing conditions of each model group.

Groups	Drugs	Dosage	Administration	Time for drug
Healthy	Saline	5mg/kg·d	Gavage	8 w
Diabetic	Saline	5mg/kg·d	Gavage	8 w
Macroangiopathy	Saline	5mg/kg·d	Gavage	8 w
SHENQI	SHENQI	14.4g/kg·d	Gavage	8 w
Yangyinyiqi	Yangyinyiqi	11.7g/kg·d	Gavage	8 w
Huoxue	Huoxue	2.7g/kg·d	Gavage	8 w
Rosiglitazone	Rosiglitazone	1.33mg/kg·d	Gavage	8 w

SHENQI compound: rehmanniaglutinosa 10g, yam yam 10g, hawthorn 10g, raw radix 15g, ginseng 15g, salvia miltiorrhiza 10g, rhubarb 6g, trichosanthin 10g; yangyinyiqi formula: ginseng 15g, Astragalus 15g, rehmannia root 10g, trichosanthin 10g, hawthorn 10g, Huai Yam 10g; v formula: salvia miltiorrhiza 10g, rhubarb 6g. SHENQI compound extract: 1.44g of raw medicine per ml of extract, produced by the Department of Pharmacy, Affiliated Hospital of Chengdu University of Traditional Chinese Medicine, batch number: 20120817; yangyinyiqi formula extract: each ml extract containing 1.17g of raw medicine per ml of extract, produced by the Department of Pharmacy, Affiliated Hospital of Chengdu University of Traditional Chinese Medicine, batch number: 20120817; huoxue formula extract: 0.27g of raw medicine per ml of extract, produced by the Department of Pharmacy, Affiliated Hospital of Chengdu University of Traditional Chinese Medicine, batch number: 20120815.

### Differential expression analysis

First, background correction and normalization were performed on the resulting full-spectrum chip data (GSE121487) using the Background Correct function in the R language limma package [48–51]. Then, control probes and low-expression probes were filtered using the normalize between Arrays function quantile normalization. Finally, the differentially expressed genes of seven datasets were identified using the lmFit and eBayes functions of the R language limma package, with default parameters, p value < 0.05.

### Co-expression analysis

Based on the differentially expressed genes in diabetes and diabetic macroangiopathy group, weighted gene co-expression network analysis was performed using the R language package WGCNA [52].

### Pivot regulators analysis

Pivot regulators are TFs or ncRNAs significantly regulated modules. A regulator was defined as pivot regulators, if the regulators interacted with at least 2 genes and the number of interactors significant enriched for each module (hypergeometric tests, P value < 0.01). ncRNA-associated interactions in mouse were exacted form RAID 2.0 database (score > 0.5) [53], involving 2310 ncRNAs, 80343 interaction relationships. Transcription factor-target entries in mouse were exacted from the TRRUST v2 database [54], a total of 827 TFs and 7,057 interactions. And drug-target relationships were exacted from the Drug bank database [55].

### Functional and pathway enrichment analysis

The function and pathway enrichment analysis of this study were performed using the David Database [47], p value < 0.05.

## Supporting information

S1 Table Comparison of weekly mean blood glucose changes in each group. Compared with the normal group, ▲P <0.05, ▲▲P <0.01. Compared with the diabetic group, ★P <0.05, ★★P <0.01. Compared with the model group, ※P <0.05, ※※P <0.01.
(XLS)

S2 Table Differentially expressed genes. (XLS)

S3 Table Enrichment results of differentially expressed gene. (XLS)

S4 Table Modular genes involved in the function and pathway. (XLS)

S5 Table Pivot prediction results for ncRNA. (XLS)

S6 Table Pivot prediction results for TF. (XLS)

S7 Table Therapeutic effects of SHENQI compound and rosiglitazone on the module. (XLS)

S8 Table Pivot prediction results for drug. (XLS)
